# In Vivo Evaluation of Demineralized Bone Matrix with Cancellous Bone Putty Formed Using Hydroxyethyl Cellulose as an Allograft Material in a Canine Tibial Defect Model

**DOI:** 10.3390/ani14202997

**Published:** 2024-10-17

**Authors:** Donghyeok Yoo, Minha Oh, Minkyung Kim, Dongbin Lee

**Affiliations:** 1 Institute of Animal Medicine, College of Veterinary Medicine, Gyeongsang National University, Jinju 52828, Republic of Korea; 2 Mbiologic LLC., 18017 Sky Park Circle, Irvine, CA 92614, USA; 3 Keunmaum Animal Medical Center, Haeundaegu, Busan 28096, Republic of Korea

**Keywords:** bone defect, bone tissue engineering, bone substitutes, canine allograft, demineralized bone matrix, hydroxyethyl cellulose

## Abstract

**Simple Summary:**

Bone defects present significant challenges in orthopedics, with alternatives to autografts commonly required owing to issues such as donor-site morbidity. Allogeneic demineralized bone matrix (DBM) is one viable option; however, handling difficulties and low mechanical strength necessitate effective carriers. Currently, DBM formulations are usually a paste or putty. This study evaluated hydroxyethyl cellulose (HEC) as a novel DBM carrier to improve clinical outcomes in a canine tibial defect model. We aimed to enhance the mechanical strength and osteogenic properties of DBM by incorporating cancellous bone or calcium phosphate. Our results suggest that combining DBM with HEC effectively promotes bone regeneration.

**Abstract:**

Demineralized bone matrix (DBM) is a widely used allograft material for bone repair, but its handling properties and retention at defect sites can be challenging. Hydroxyethyl cellulose (HEC) has shown promise as a biocompatible carrier for bone graft materials. This study aimed to evaluate the efficacy of DBM combined with cancellous bone putty formed using HEC as an allograft material for bone regeneration in a canine tibial defect model. Experiments were conducted using dogs with proximal tibial defects. Four groups were compared: empty (control group), DBM + HEC (DH), DBM + cancellous bone + HEC (DCH), and DBM + cancellous bone + calcium phosphate + HEC (DCCH). Radiographic, micro-computed tomography (CT), and histomorphometric evaluations were performed 4 and 8 weeks postoperatively to assess bone regeneration. The Empty group consistently exhibited the lowest levels of bone regeneration throughout the study period, indicating that DBM and cancellous bone with HEC significantly enhanced bone regeneration. At week 4, the DCCH group showed the fastest bone regeneration on radiography and micro-computed tomography. By week 8, the DCH group showed the highest area ratio of new bone among all experimental areas, followed by the DH and DCCH groups. This study demonstrated that HEC significantly enhances the handling, mechanical properties, and osteogenic potential of DBM and cancellous bone grafts, making it a promising carrier for clinical applications in canine allograft models. When mixed with allograft cancellous bone, which has high porosity and mechanical strength, it becomes a promising material offering a more effective and reliable option for bone repair and regeneration.

## 1. Introduction

Bone defects represent a significant clinical challenge, with advanced orthopedic techniques often involving the use of autogenous, allogeneic, or xenografts required to ensure proper healing [1]. Although autografts are considered the gold standard for bone defect treatment, their limitations, such as donor-site morbidity, immune responses, ineffective integration, and limited availability, have resulted in the development and use of many alternative substitutes [2,3]. Among these alternatives, allografts, particularly demineralized bone matrix (DBM), were among the first to be developed [3]. DBM is derived from allograft bone sources, which are processed to remove mineral components while retaining the organic factors of the bone, such as growth factors, collagen, and other proteins integral to bone regeneration [4,5]. As such, DBM has osteoinductive properties due to growth factors such as bone morphogenetic proteins, which stimulate the surrounding mesenchymal stem cells to differentiate into osteoblasts to foster bone formation and healing. These features make DBM an effective option for bone regeneration, as it supports new bone growth and provides a scaffold for osteoprogenitor cells [3]. However, DBM has low mechanical strength and can be difficult for clinicians to handle because of its particulate nature following demineralization. DBM powder or particles may not remain secure at the defect site and can be easily displaced by irrigation and blood flow during surgery [2]. As such, DBM is typically combined with other allografts or carriers, which act as infillers to enhance its stability and effectiveness during surgical procedures.

In the past, efforts have been made to enhance the bone-healing response of DBM by combining it with various graft materials. These attempts sought to address the limitations of DBM, as the rapid release of osteoinductive factors can result in an inconsistent bone healing response throughout the healing process [6]. Many DBM carriers with different characteristics have previously been studied and commercialized. Most DBM products currently incorporate carrier materials, such as glycerin and hyaluronic acid, which may not guarantee stable retention or reliable osteogenic efficacy at the defect site [2,7,8]. To date, no single carrier has yet satisfied all of these requirements, raising questions about the clinical effectiveness, future opportunities, and optimal use of these products [8,9].

Hydroxyethyl cellulose (HEC) is a water-soluble polymer derived from cellulose with gel-like properties, with excellent biocompatibility, biodegradability, and hydrophilicity, with no toxicity, making it a versatile and valuable component in many applications [10,11]. HEC has been used as a scaffold in previous in vitro studies, which have reported an associated increase in the attachment and proliferation of human osteosarcoma cells and human fetal osteoblast cells, demonstrating that HEC is an ideal scaffold for bone cell culture [12,13]. Given these properties, HEC has significant potential as a carrier for DBM, meeting the many conditions required for an ideal carrier.

In this study, we assessed HEC as a carrier of DBM during allograft procedures, specifically focusing on small bone defects. The aim was to determine which combination of DBM and HEC, when supplemented with materials such as allogeneic cancellous bone or calcium phosphate, would optimally facilitate bone regeneration. As such, the overall aim of this study was to provide new insights into improving bone graft outcomes using HEC, particularly in canine allograft models, where its application has not been extensively studied. We hypothesize that combining demineralized bone matrix (DBM) with hydroxyethyl cellulose (HEC) as a carrier, supplemented with allogeneic cancellous bone and/or calcium phosphate, will enhance bone regeneration in a canine tibial defect model.

## 2. Materials and Methods

This study was conducted as a prospective, randomized, blinded clinical trial to evaluate the efficacy of demineralized bone matrix (DBM) combined with cancellous bone putty formed using hydroxyethyl cellulose (HEC) as an allograft material for bone regeneration in a canine tibial defect model.

### 2.1. Animal Preparation

In this study, eight intact male mongrel dogs weighing 25–30 kg and aged 10–12 months were included as the study objects. Considering the variations in body size, the dogs’ body condition score (BCS) was assessed, with all animals scoring between 4–5 on a 9-point scale. Dogs were excluded from the experiment if they exhibited any signs of illness, such as fever, lethargy, or gastrointestinal distress during the seven-day quarantine and purification period. Additionally, dogs that experienced significant weight loss exceeding 5% of their body weight or displayed abnormal behavior, such as excessive anxiety or aggression, were also excluded. Physical examinations were conducted to ensure that there were no clinical abnormalities, including respiratory, cardiovascular, or musculoskeletal issues, and laboratory tests were performed to rule out infections, anemia, or other systemic health conditions. Dogs with a previous history of surgical interventions or injuries that could affect the study’s outcomes were also excluded. Only dogs that met all these criteria and were deemed clinically healthy were included in the study. Two defect sites were established on the proximal tibias of each dog. The following materials were used to fill each fault spot: Site 1 (L-1) of the left tibial defect was left empty, whereas site 2 (L-2) was treated with DBM in addition to HEC (DH). The DBM, cancellous bone, and HEC (DCH) were implanted at the right tibial defect site 1 (R-1), whereas the DBM, cancellous bone, calcium phosphate, and HEC (DCCH) were placed at site 2 (R-2).

### 2.2. Preparation of Experimental Materials

The DH group underwent implantation with DBM and HEC (DynaFuse; Veteregen); the DCH group with cancellous chips mixed with DBM and HEC (NatraFuse; Veteregen); and the DCCH group with cancellous chips and calcium phosphate mixed with DBM and HEC (PureFuse; Veteregen). All products were made from bone aseptically harvested from animals euthanized for reasons unrelated to tissue transplantation after obtaining the written consent of the owners. The production of demineralized bone matrix (DBM) putty begins with the acquisition of raw materials, typically donated canine bones such as the ulna, femur, and tibia. These bones are thoroughly washed to remove impurities like blood, fat, and soft tissues. Following this, the bones undergo dehydration using alcohol to remove moisture, which could be a potential source of infection. Once dehydrated, the bones are subjected to a defatting process. This involves mixing the bones in a shaker with ether or other reagents to eliminate any remaining fat content, reducing the risk of immune rejection. After the bones are defatted, they are ground into a specific size. The next step is demineralization, where strong acids such as hydrochloric acid (HCl) or acetic acid are used to dissolve the minerals, primarily calcium, from the bone. This process preserves the organic components of the bone matrix, such as collagen and growth factors, which are crucial for bone regeneration. Finally, the DBM is freeze-dried to reduce its moisture content. The cancellous bone particles, demineralized bone matrix, and calcium phosphate particles all ranged from 200–750 µm, with the latter having a 20% porous structure. Hydroxyethyl cellulose and glycerin were combined to prepare an aqueous solution (HEC solution) with a concentration of 5% HEC and 60% glycerin. In the bone-graft materials used in the experiment, the HEC solution comprised 70% of the total weight, with the remaining components constituting 30%. The DBM to cancellous bone ratio in the DCH group was 6:4. In the DCCH group, the proportions of DBM, cancellous bone, and calcium phosphate were balanced at 6:2:2. The specific composition ratios for each group are listed in Table 1.

### 2.3. Anesthesia and Perioperative Management

All dogs were fasted for 12 h with free access to water until 2 h before the procedure. Premedication prior to intervention included tiletamine-zolazepam (10 mg/kg, IM, Zoletil 100^®^, Virbac Animal Health, Carros, France), xylazine (1 mg/kg, IM, Rompun^®^, Bayer Korea, Seoul, Republic of Korea) and butorphanol (0.2 mg/kg, IV, Butophan injection^®^, MYUNGMOON PHARM, Seoul, Republic of Korea). For induction, half the quantity of Zoletile was injected intravenously. Anesthesia following endotracheal intubation using an endotracheal tube was maintained via inhalation anesthesia comprising oxygen and isoflurane (TerrelTM, Piramal Critical Care, Bethlehem, PA, USA) with the vaporizer setting at 2.0–3.0% to achieve a target minimum alveolar concentration (MAC) of approximately 1.3%, which was subsequently adjusted as needed based on continuous monitoring of the patient’s response to surgical stimulation. During surgery, the anesthetist continuously monitored the dogs’ heart rate, blood pressure, saturation pulse oxygen, end-tidal carbon dioxide, respiratory rate, and temperature using a monitoring device (Carestation 750 Anesthesia Delivery system, GE HealthCare, Wauwatosa, WI, USA). After the procedure, each dog was placed in a cage and provided with water and dry food. On the day of surgery, the dogs were kept in their cages for rest and recovery. Starting the following day, they were taken on light leash walks twice a day for approximately 10 min each. After two weeks, only excessive movements were restricted. Enrofloxacin (10 mg/kg, IM, once a daily, Baytril^®^, Elanco Animal Health, Greenfield, IN, USA) and meloxicam (0.1 mg/kg, SC, once daily, Metacam^®^, Boehringer Ingelheim, Duluth, GA, USA) were administered for 3 days, and the surgical site was disinfected with 0.1% chlorhexidine every day until the stitches were removed. Only minor complications, such as bruising, were observed in the dogs, and they resolved within a few days post-surgery without the need for any additional treatment [14].

### 2.4. Surgical Procedure

An IV catheter was placed in the cephalic vein, and Hartmann’s solution, an isotonic crystalloid, was administered at the maintenance fluid rate (5 mL/kg/hr). The surgical site was prepared aseptically by clipping a wide area of the hair and disinfecting the skin with a chlorhexidine-alcohol solution. The surgical site was prepared aseptically, and the dogs were placed in a dorsal recumbent position. An incision was made on the skin approximately 3 cm along the medial side of the tibia, and the subcutaneous fat and superficial fascia were severed underneath the skin incision. The medial metaphysis of the bone was incised to expose the bone, and the caudal sartorius, popliteus, and cranial tibialis muscles were retracted. Two defect sites were generated in the metaphyseal bone using a high-speed air drill equipped with a 5 mm bur at a depth of 3 mm, with continuous saline irrigation. Each hole was created with minimum space (2.5 cm) on both the proximal and distal aspects of the tibial metaphysis. The fault spots produced were filled with test materials using a spatula. The muscle and subcutaneous tissues were subsequently sutured with 3–0 polydioxanone, and the skin was finally closed using a 3–0 non-absorbable suture. The same procedure was performed for the contralateral tibia [15,16].

### 2.5. Postoperative Assessment

#### 2.5.1. Radiographic Evaluation

Radiographic examinations were performed at 0, 4, and 8 weeks postoperatively. Mediolateral views of the affected limb were centered on the tibial metaphyseal defect. The images obtained at each time point were independently evaluated by two blinded observers, who were unaware of the experimental groups. Scores were assigned based on the following criteria: a score of 0 indicates that there is no bone formation in the defect, 1 indicates that bone just extends into the defect, 2 indicates that the bone substantially bridges the cortical defect, 3 indicates that the bone has fully bridged the cortex without a significant callus, and 4 indicates that the bone has fully bridged the cortex with a distinct overlying callus. The scores for each defect site were calculated based on the evaluations of each observer according to the method described above. The scoring method used in this study was developed based on the Radiographic Union Score for Tibial fractures (RUST), with modifications to fit the specific characteristics of our tibial metaphyseal defect model [17].

#### 2.5.2. Micro-Computed Tomographic (CT) Evaluation

At 4 weeks post-operative, two dogs were sacrificed, followed by an additional two at 8 weeks post-operation, to obtain the tibia bones for micro-CT evaluation. After capturing images of the defect site with a micro-CT scanner (SkyScan1173; Bruker-CT, Kartuizersweg 3B 2550 Kontich, Kontich, Belgium), we obtained high-resolution images by rotating the images 180 degrees using SkyScan1173 control software (Ver. 1.6, Bruker-CT). The shooting conditions were: 90 kVp tube voltage, 88 μA tube current, 1 mm aluminum filtration, with an exposure time of 500 ms, (2240 × 2240) pixels, pixel size of 29.83 μm, and a rotation angle of 0.3°. Images were rotated 180° to obtain a total of 800 high-resolution images. For section reconstruction, an image of 2240 × 2240 Pixel was obtained using Nrecon (Ver. 1.7.0.4, Bruker-CT), and the axis was aligned using a Dataviewer (Ver. 1.5.1.2, Bruker-CT). As an analysis program, a Ct Analyzer (Ver. 1.14.4.1, Bruker-CT) was used to separate and analyze the bone trabeculae and marrow cavities in each image.

#### 2.5.3. Histomorphometric Evaluation

The extracted bone samples were fixed in 10% buffered neutral formalin (Sigma-Aldrich, St. Louis, MO, USA) for one week, followed by decalcification with 5% nitric acid. The specimens were then serially dehydrated in ethanol and embedded in paraffin after cleansing with xylene. They were then sectioned at 4 µm using an automated rotary microtome (Leica RM2255, Leica Biosystems, Nußloch, Germany) and stained with Harris’s alum Hematoxylin and Eosin (Sigma-Aldrich, USA). The degree of bone regeneration was assessed through analysis of the tissue morphology of samples from the experimental groups treated with the test materials. For histomorphometric measurements, a digital image was obtained using Pannoramic 250 Flash III of 3D Histech (No. 3 Ov Street Budapest, Budapest, Hungary), after which images were acquired using Caseviewer (ver. 2.1, 3D Histech, Budapest, Hungary). In addition, for histomorphometric measurement, 3 points were specified using the curve of autocad to confirm the analysis location, and Image Pro plus ^®^ (Ver. 7.0, MediaCybernetics, Inc., Rockville, MD, USA) was used as the image analysis software.

### 2.6. Statistical Analysis

A priori power analysis to investigate sample size was calculated by using G*Power (version 3.1.9.7, Heinrich–Heine University Düsseldorf, Düsseldorf, Germany), and 8 samples in each group were suggested for the power of 80% and α level of 0.05.

All experimental data are expressed as the mean ± standard deviation (SD). Kruskal-Wallis test and post hoc Dunn’s test were used to compare radiographic scores between four groups. Regarding the area and ratio of new bone regeneration, the Shapiro-Wilk test was used to determine the normal distribution of the data. According to the results, the Kruskal-Wallis test was used to compare the area of new bone regeneration through micro-computed tomographic evaluation and post hoc Dunn’s test was conducted to determine statistical differences between each group. One-way ANOVA was performed to compare the new bone regeneration ratio through histomorphometric evaluation and post hoc Bonferroni’s test was used. *p* values less than 0.05 were considered statistically significant. All analyses were performed using GraphPad Prism (version 8.0.2, GraphPad Software, San Diego, CA, USA) and IBM SPSS Statistics (version 27.0, IBM Corp., Armonk, NY, USA).

## 3. Results

### 3.1. Radiographic Evaluation

**Figure 1:**
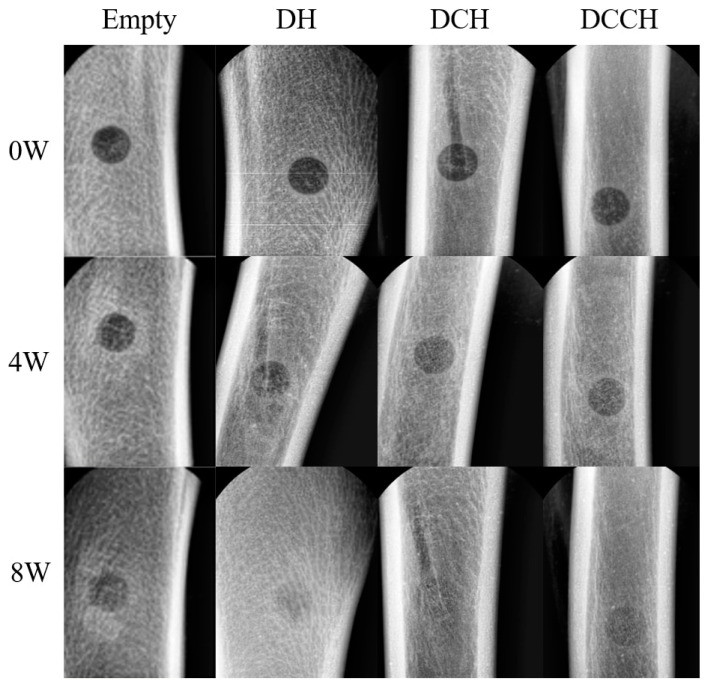
Representative radiographic images of the Empty, DH, DCH, and DCCH groups at 0, 4, and 8 weeks post-implantation. At 0 weeks, all groups exhibited similar radiographic appearance due to low radiopacity. At 8 weeks, new bone formation was evident, with minimal bone defects visible, in all groups except the Empty group.

We implanted DH, DCH, and DCCH grafts in a dog tibia defects for 4 and 8 weeks to examine in vivo bone development (Table 2, Figure 1). All groups exhibited a trend towards enhanced bone growth according to the results assessed at both timepoints following test material engraftment. Furthermore, all treatment groups showed improved bone growth compared to the Empty (L-1) group. At week 4, the DCCH group achieved the highest score, indicating a high radiodensity, whereas the empty group received the lowest score. By week 8, both the DH and DCH groups received scores of 4 out of 4 based on the visual assessments by both observers, who noted complete continuity of the radiographic cortex and clear evidence of callus formation. In contrast, the DCCH group scored slightly lower at 3.5. The Empty group received a distinctly lower score of 1.8, which was markedly different from those of the other groups.

### 3.2. Micro-Computed Tomographic Evaluation

**Figure 2:**
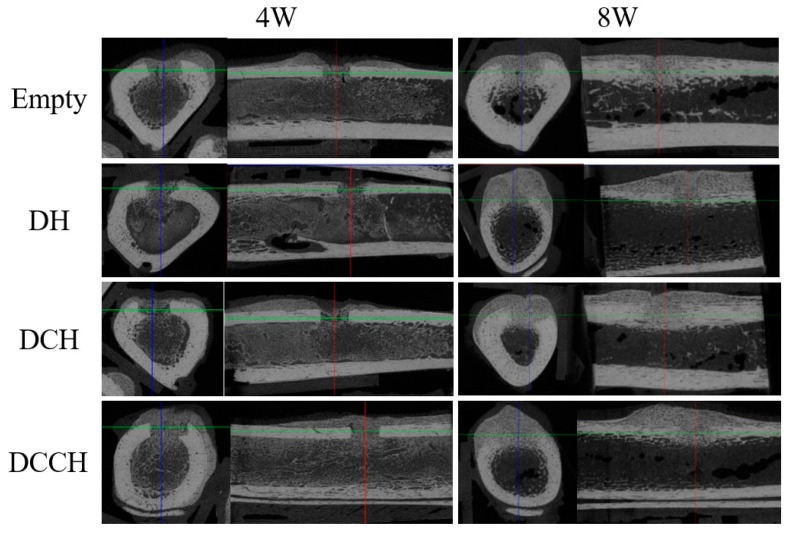
Micro-computed tomographic evaluation of each group at 4 and 8 weeks, as shown in the transverse and sagittal views. Comparable development of both the external and the internal callus was observed in the DH, DCH, and DCCH groups.

New bone formation in various bone transplants was assessed through a quantitative examination of the new bone area in the entire tissue. In all groups, bone regeneration showed better outcomes in the eighth week than in the fourth week (Table 3 and Figure 2). Comparable levels of new bone regeneration were further observed at week 8 in all three experimental groups containing HEC with DBM, and at both weeks, the new bone regeneration area was approximately 36% larger than that in the empty group. Furthermore, compared with the other groups, the DCCH group showed a higher rate of new bone regeneration at 4 weeks; however, it did not attain the best results for bone regeneration at 8 weeks. None of the data showed statistical significance between groups.

### 3.3. Histomorphometric Evaluation

**Figure 3:**
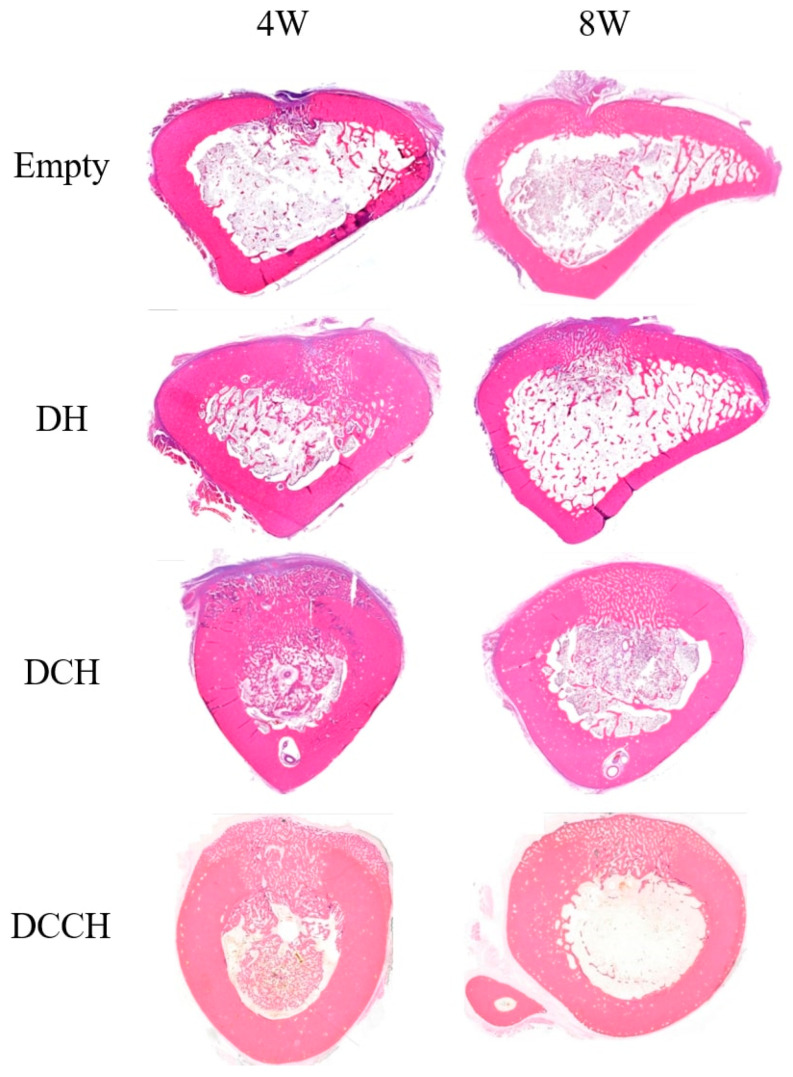
Histological images stained with hematoxylin and eosin showing representative grafted regions and union sites in each group at 4 and 8 weeks (magnification ×40).

**Figure 4:**
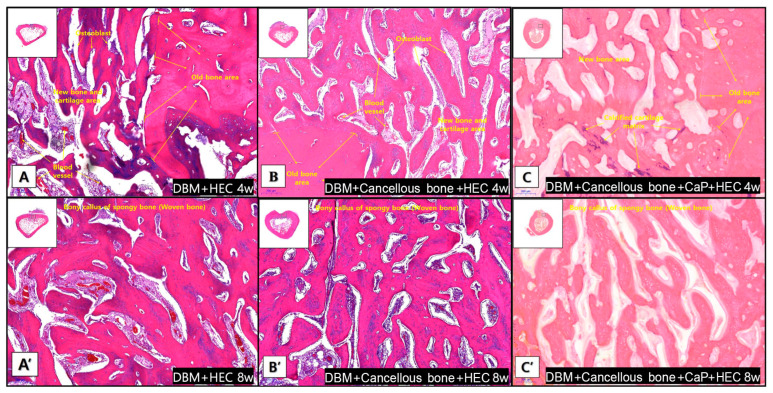
Representative histological images of the grafted region and union sites in each group at 4 and 8 weeks stained with hematoxylin and eosin (magnification ×100). (**A**–**C**) At week 4, the boundary between the old bone area and the new bone area was clearly demarcated. Osteoblasts and blood vessels are present in the new bone area. (**A**’–**C**’) In week 8, woven bone formation is observed in all bones.

The implanted grafts were stained with hematoxylin and eosin to evaluate new bone formation. Histomorphometric analysis revealed superior bone regeneration in the groups treated with HEC and DBM compared to the empty group at both 4 and 8 weeks postoperatively (Table 4, Figure 3 and Figure 4). Specifically, the DCH group exhibited the highest degree of regeneration at 4 weeks, followed by the DCCH group. At the 8-week mark, DCH continued to exhibit the most substantial bone regeneration among all groups, followed by DH and DCCH.

## 4. Discussion

Currently, most DBM products commonly used in humans are created in the form of paste or putty combined with various carriers, as implantation of DBM alone is associated with several challenges, such as low mechanical strength and rapid resorption. Combining DBM with other materials enhances its versatility, expands its capabilities for bone repair, and addresses its inherent weaknesses [3,8,18]. Additionally, DBM powders or particles can be unstable and prone to displacement during surgery, while preformed materials may not fit well into the defect sites, potentially causing nonunion or delayed healing [8]. Consequently, extensive research on DBM carriers has been conducted in recent years. However, most studies on DBM and carriers have been conducted in vitro, while in vivo studies have predominantly used rats and rabbits, with only a few studies involving dogs [8,19]. This study, therefore, aimed to determine the effectiveness of DBM using allogeneic grafts in canines.

The carrier medium in DBM products typically enhances handling but may dilute the active DBM components, potentially reducing the product’s osteoinductive capacity. An ideal carrier for bone formation should have excellent biocompatibility without inducing immune reactions, a biodegradation rate matching bone healing, suitable viscosity, and flexibility for shaping and secure placement [3,8,20]. In previous studies involving canines, DBM was combined with carriers such as glycerol, calcium-based putty, and platelet-rich plasma; however, no studies have yet used HEC as a carrier in canines [21,22]. Cellulose-based composite carriers fulfill many of these criteria and have been used in a variety of bone tissue engineering studies [23]. Using HEC in conjunction with DBM can help to overcome its drawbacks, including reduced bone density and mechanical strength due to demineralization, by providing a supportive scaffold that facilitates graft integration and osteogenesis. DBM further enhances bone regeneration through its osteoinductive properties [8,23]. Our results confirmed that DBM combined with HEC effectively promoted bone regeneration without inducing complications. Consequently, the DH, DCH, and DCCH groups exhibited superior bone regeneration compared with the empty group in all experimental evaluations, including radiography, micro-CT, and histology. Additionally, the putty-like DBM demonstrated excellent handling properties and precise application to defects, consistent with the findings of studies using glycerol as the carrier [21].

Owing to its low structural strength, DBM is predominantly used in structurally stable environments. However, combining it with excipients such as hydroxyapatite, allogeneic corticocancellous bone, or bone marrow aspirate can enhance its handling characteristics and mechanical properties [24]. For example, one study in humans demonstrated that the use of allogeneic cancellous bone in combination with DBM led to superior bone regeneration compared to DBM alone [25]. Therefore, in the present study, we aimed to evaluate the effects of adding cancellous bone and/or calcium phosphate to DBM+HECs and compare these effects with those in the DH group in canines.

Autologous cancellous bone is widely recognized as an effective bone substitute because of its high concentration of osteoblasts, bone morphogenic proteins, and mesenchymal stem cells. These components all play pivotal roles in facilitating osteointegration and providing essential structural support, thereby fostering the formation of a resilient bone matrix at the defect site [26,27]. However, autologous cancellous bone has several limitations, such as limited quantity, increased surgical time, and donor site morbidity, which can complicate its use in surgical procedures [28]. Commercially available allograft products can mitigate these challenges. Although allografts are generally believed to be inferior to autografts in terms of functionality, one study comparing autogenous and allogenous cancellous bone grafts in human patients found that both groups achieved equivalent radiographic and clinical outcomes [29]. This study also identified several advantages of using allogenous cancellous bone over autogenous cancellous bone, including a lack of concern regarding donor site pain and a theoretically unlimited supply. Furthermore, one retrospective case study of allograft products in dogs that used DBM in combination with allogeneic cancellous powder and cortical chips indicated that comparable results could be achieved without adverse effects associated with autologous cancellous bone harvesting [22]. Consequently, we evaluated allogeneic cancellous bone for practical clinical applications and sought to enhance its usability and functionality by incorporating HEC as a carrier. The DCH group achieved the most notable results in radiographic, micro-CT, and histological assessments at 8 weeks.

Calcium phosphate is highly valued owing to its osteoconductive properties, ease of handling, radiopacity, low immunogenicity, minimal risk of disease transmission, slow resorption, and strong mechanical properties [3,30]. In this study, the inclusion of calcium phosphate led to the most significantly promising results in the radiographic and micro-CT evaluations at 4 weeks, with the DCCH group showing the fastest bone regeneration. This suggests a possible synergistic effect between calcium phosphate and other osteoinductive components, potentially due to the presence of growth factors at four weeks. However, the DCCH group showed lower histological scores than the DCH group at both 4 and 8 weeks and even lower bone regeneration rates than the DH group at 8 weeks. Although the DCCH group showed excellent bone regeneration ability at the 8-week evaluation, the DCH group overall yielded the best overall results. This discrepancy may be due to the lower porosity of the calcium phosphate used (approximately 20%). Optimal porosity is a crucial requirement for ideal carriers. Indeed, various studies have demonstrated that osteoconduction requires a high porosity of bone grafts (>50%), emphasizing the significance of both micro- and macroporosity in the osseointegration of calcium phosphates [31,32].

This study had several limitations. First, it did not include a group using only DBM without any carriers or additives or a group using only HEC without DBM, which made it difficult to isolate the specific effects of HEC and other additives on bone regeneration. Second, the study had a relatively small sample size, which could have impacted the statistical power and robustness of the conclusions. Third, our study focused on small bone defects. In larger or more critical defect models, such as weight-bearing bone defects, the mechanical stability of the graft material becomes more important and may yield different results. Fourth, while physiological parameters were continuously monitored during anesthesia, they were not systematically recorded. This limitation is significant, as prolonged anesthesia can lead to surgical stress, potentially affecting osteosynthesis and increasing the risk of bone nonunion [33,34]. The lack of such data in this study makes it difficult to assess the impact of anesthesia duration on bone regeneration outcomes. Future studies should include the systematic recording of physiological parameters during anesthesia to better understand these effects. Additionally, the study did not evaluate postoperative pain using specific scales or monitors, such as the PTA index, which could have provided more insights into the animals’ recovery experience. The absence of biomechanical tests during movement limited the evaluation of clinical recovery. Furthermore, the lack of measurement of blood immunological biomarkers, such as stress markers, meant that valuable data on the degree of surgical stress and the effectiveness of DBM in the applied model were not obtained. Finally, the evaluation period was limited to 4 and 8 weeks postoperatively. Long-term studies are therefore required to assess the durability and quality of bone regeneration over extended periods.

Despite these limitations, the results indicated that DBM and cancellous bone with HEC significantly enhanced bone regeneration compared to empty defects, regardless of the addition of additives. HEC’s biocompatibility, controlled-release properties, viscosity control, and potential to improve mechanical properties make it a promising carrier for bone regeneration. Notably, no adverse events were reported when the HEC were used as carriers, highlighting their suitability for clinical use.

## 5. Conclusions

Overall, this study demonstrated that combining DBM and cancellous bone with HEC significantly improved the functionality of bone graft materials compared with an empty control. HEC enhances the handling characteristics, making it easier to apply to defect sites. Adding cancellous bone to DBM with HEC further improved the osteogenic potential of the grafts, leading to superior bone regeneration. Despite some study limitations, this study showed the strong potential of HEC as an excellent carrier for DBM, offering significant benefits, such as a lack of side effects and ease of use, making it suitable for clinical applications. This is the first study to use HEC as a carrier for DBM in canine allograft models, while comparisons of different combinations that use cancellous bone-induced superior osteogenesis.

## Figures and Tables

**Table 1:** Percentage compositions of bone graft material components in each group (%).

Bone Tissue	DH	DCH	DCCH
DBM 100% (*w*/*w*)	30%	-	-
DBM 60% (*w*/*w*) + Cancellous bone 40% (*w*/*w*) mixture	-	30%	-
DBM 60% (*w*/*w*) + Cancellous bone 20% (*w*/*w*) + Calcium phosphate 20% (*w*/*w*) mixture	-	-	30%
5% HEC solution	70%	70%	70%
Total	100%	100%	100%

DBM, demineralized bone matrix; HEC, hydroxyethyl cellulose; DH, DBM + HEC; DCH, DBM + cancellous bone + HEC; DCCH, DBM + cancellous bone + calcium phosphate + HEC.

**Table 2:** Radiographic scores (0–4) evaluated by two blind observers (Mean ± SD).

	4 Week	8 Week
Empty	0.3 ± 0.5 ^a,b^	1.8 ± 0.5 ^c,d^
DH	1.6 ± 0.7	4.0 ± 0.0 ^c^
DCH	2.0 ± 1.1 ^a^	4.0 ± 0.0 ^d^
DCCH	2.4 ± 0.7 ^b^	3.5 ± 0.6

DH, DBM + HEC; DCH, DBM + cancellous bone + HEC; DCCH, DBM + cancellous bone + calcium phosphate + HEC. ^a^ *p* < 0.01, ^b^ *p* < 0.001, ^c,d^ *p* < 0.05.

**Table 3:** The area of new bone regeneration obtained through micro-computed tomographic evaluation (Mean ± SD, mm^3^).

	4 Weeks	8 Weeks
Empty	50.88 ± 3.33	54.04 ± 2.52
DH	59.92 ± 1.80	78.14 ± 2.42
DCH	56.80 ± 11.22	80.79 ± 2.27
DCCH	73.64 ± 0.36	80.23 ± 2.54

DH, DBM + HEC; DCH, DBM + cancellous bone + HEC; DCCH, DBM + cancellous bone + calcium phosphate + HEC. No statistical significance was analyzed between groups.

**Table 4:** The new bone regeneration ratio obtained through histomorphometric evaluation (Mean ± SD, %).

	4 Week	8 Week
Empty	29.63 ± 11.95 ^a,b^	42.79 ± 9.42 ^c,d,e^
DH	48.98 ± 18.44	69.36 ± 5.14 ^c^
DCH	63.36 ± 8.81 ^a^	77.56 ± 3.90 ^d,f^
DCCH	61.01 ± 7.83 ^b^	62.89 ± 1.90 ^e,f^

DH, DBM + HEC; DCH, DBM + cancellous bone + HEC; DCCH, DBM + cancellous bone + calcium phosphate + HEC. ^a,b,f^ *p* < 0.05, ^e^ *p* < 0.01, ^c,d^ *p* < 0.001.

## Data Availability

The data presented in this study are available upon request from the corresponding author.
